# Bone Metastasis and Immune Checkpoint Inhibitors in Non-Small Cell Lung Cancer (NSCLC): Microenvironment and Possible Clinical Implications

**DOI:** 10.3390/ijms23126832

**Published:** 2022-06-20

**Authors:** Alessandro Del Conte, Elisa De Carlo, Elisa Bertoli, Brigida Stanzione, Alberto Revelant, Manuela Bertola, Michele Spina, Alessandra Bearz

**Affiliations:** 1 Dipartimento di Oncologia Medica, Centro di Riferimento Oncologico di Aviano (CRO) IRCCS, 33081 Aviano, Italy; elisa.decarlo@cro.it (E.D.C.); elisa.bertoli@cro.it (E.B.); brigida.stanzione@cro.it (B.S.); manuela.bertola@cro.it (M.B.); mspina@cro.it (M.S.); abearz@cro.it (A.B.); 2 Department of Medicine (DAME), University of Udine, 33100 Udine, Italy; 3 Dipartimento di Radioterapia, Centro di Riferimento Oncologico di Aviano (CRO) IRCCS, 33081 Aviano, Italy; alberto.revelant@cro.it

**Keywords:** bone metastasis, immunotherapy, immune checkpoint inhibitors, microenvironment, non-small cell lung cancer (NSCLC)

## Abstract

Patients with non-small cell lung cancer (NSCLC) develop bone metastasis (BoM) in more than 50% of cases during the course of the disease. This metastatic site can lead to the development of skeletal related events (SREs), such as severe pain, pathological fractures, spinal compression, and hypercalcemia, which reduce the patient’s quality of life. Recently, the treatment of advanced NSCLC has radically changed due to the advent of immunotherapy. Immune checkpoint inhibitors (ICI) alone or in combination with chemotherapy have become the main therapeutic strategy for advanced or metastatic NSCLC without driver gene mutations. Since survival has increased, it has become even more important to treat bone metastasis to prevent SRE. We know that the presence of bone metastasis is a negative prognostic factor. The lower efficacy of immunotherapy treatments in BoM+ patients could be induced by the presence of a particular immunosuppressive tumor and bone microenvironment. This article reviews the most important pre-clinical and clinical scientific evidence on the reasons for this lower sensitivity to immunotherapy and the need to combine bone target therapies (BTT) with immunotherapy to improve patient outcome.

## 1. Introduction

Lung cancer is the leading cause of cancer-related death [1]. In recent years, there has been an improvement in cancer biology and immune system knowledge. In particular, the treatment of non-small cell lung cancer (NSCLC) has radically changed due to the introduction of new molecules with a molecular target and the advent of immunotherapy. Immune checkpoint inhibitors (ICIs), which target programmed-death 1 (PD1) and PD-ligand (PD-L1), either used alone or in combination with chemotherapy have become the main therapeutic strategies for advanced or metastatic NSCLC without driver gene mutations [2,3]—since progression free survival (PFS) and overall survival (OS) has improved. Nivolumab, atezolizumab, and pembrolizumab are recommended options for patients who progress after platinum-doublet chemotherapy. The Food and Drug Administration (FDA) approved nivolumab for this indication in October 2015 and atezolizumab in October 2016. Pembrolizumab received FDA approval for this indication in October 2015 with a limitation for PD-L1 positive tumors (with the accompained diagnostic IHC 22C3 pharmaDX test). As of today, pembrolizumab alone is the standard first-line therapy for patients with PD-L1 expression >50% (FDA approval in October 2016), whereas pembrolizumab plus platinum-base chemotherapy is the treatment of choice for patients with PDL-L1 < 50% (FDA approval for non-squamous NSCLC in May 2017 and for squamous in October 2018). Recently (May 2020) nivolumab plus ipilimumab, given with two cycles of platinum-doublet chemotherapy, was approved by the FDA as a first-line treatment for metastatic NSCLC regardless of histology and PD-L1 expression.

Recently, some clinical factors, such as performance status and metastatic sites, emerged as potential predictors for immunotherapy efficacy [4,5,6]. In this narrative review, we pointed out the impact of bone metastatic site.

The incidence of bone metastasis in NSCLC varies—according to the studies taken into consideration—ranging from 20% to more than 60%. Thanks to the improvement of diagnostic techniques (e.g., PET-CT scan) associated with increased survival, the incidence of bone metastasis seems to be increased. In fact, 20–30% of NSCLC patients have bone metastasis at diagnosis and a further 35–40% of cases develop bone metastasis during the course of their disease [7,8]. We know that this metastatic site can lead to the development of skeletal related events (SREs), such as severe pain, pathological fractures, spinal compression, and hypercalcemia, all of which reduce the patient’s quality of life and performance status [9]. Bone metastasis usually indicates a poor prognosis for patients with lung cancer [10].

In the last decades, evidence has been published that indicates that bone marrow also functions in regulating the immune system and trafficking immune cells (regulatory T cells, T cells, B cells, dendritic cells, natural killer T cells, myeloid-derived suppressor cells, and mesenchymal stem cells) [11]. Bone marrow, therefore, can be considered an immune system regulator and could potentially influence the response to immunotherapy. This is the new concept of osteoimmuno-oncology (OIO), which refers to interactions between bone, immune, and tumor cells in the bone metastatic microenvironment [12]. 

To our knowledge, however, none of the previous randomized control trials have ever evaluated the impact of bone involvement in patients treated with immune checkpoint inhibitors nor stratified patients based on bone metastasis. Only about 1% (6/561) of publications with approved immunotherapies in breast, prostate, lung cancer, and melanoma patients report results on bone metastasis. Thus, the impact of BoM on ICI treatment has remained poorly studied. It was found that the tumor and bone microenvironment play an important role. Several studies [6,13,14,15] suggest that bone involvement may be a negative prognostic factor and the presence of BoM could be predictive of a poor response to ICIs.

In the last few years, it has also been hypothesized that the interaction between the tumor, the immune system, and the bone may occur through the presence of extracellular vesicles (EVs) that carry information through the bloodstream. 

The aims of this narrative review are: (1) describe the biological theories concerning the microenvironment of bone metastasis and the interaction between bone, the immune system, and neoplastic cells; (2) discuss the published clinical data of patients with NSCLC and bone metastasis treated with immunotherapy (alone or in combination with chemotherapy and/or bone-targeted therapy); and (3) discuss new perspectives in the field of osteoimmunoncology.

## 2. Bone Metastasis and Microenvironment 

To understand the new biological theories underlying NSCLC bone metastasis and the role of the microenvironment, we carefully selected articles—principally, pre-clinical review articles—on the PubMed search engine using the following terms: “bone metastasis and microenvironment”, “bone metastasis in lung cancer”, “osteoimmunoncology”, “immune checkpoint inhibitors and bone metastasis”, “osteoimmunology”, “crosstalk bone and immune cells”, and “extracellular vesicles and bone metastasis”.

Bone has a special immune microenvironment that is different from that of other organs. Pre-clinical studies have shown that bone is a particularly immunocompromised area. Most of the immune cells in the bone marrow are unable to control the proliferation of cancer cells. This is due to the presence of numerous immature and inhibitory immune cells in the premetastatic niche [11,16]. Furthermore, inside the bone marrow, the proportion of T cells is less than 5% (in peripheral blood 45–75%) and natural killer cells represent only 1–2% of lymphocytes.

About 40% of non-cytotoxic immune cells are regulatory T cells (Tregs) [17]. Other important cells with immunosuppressive activity are present in bone, such as myeloid-derived suppressor cells (MDSCs), which inhibit CD4 + T cells, CD8 + T cells, and NK cells [18]. In short, bone can be considered an immuno-privileged niche for disseminated cancer cells.

We know that the differentiation of pre-osteoblasts into osteoclasts occurs through cytokines derived from the immune system, such as macrophage colony-stimulating factor (M-CSF), interleukins (IL), transforming grow factor beta (TGF-beta), prostaglandins, and interferon gamma. Conversely, bone cells regulate immune cells by creating the so-called “endosteal niche” [19,20].

**Figure 1:**
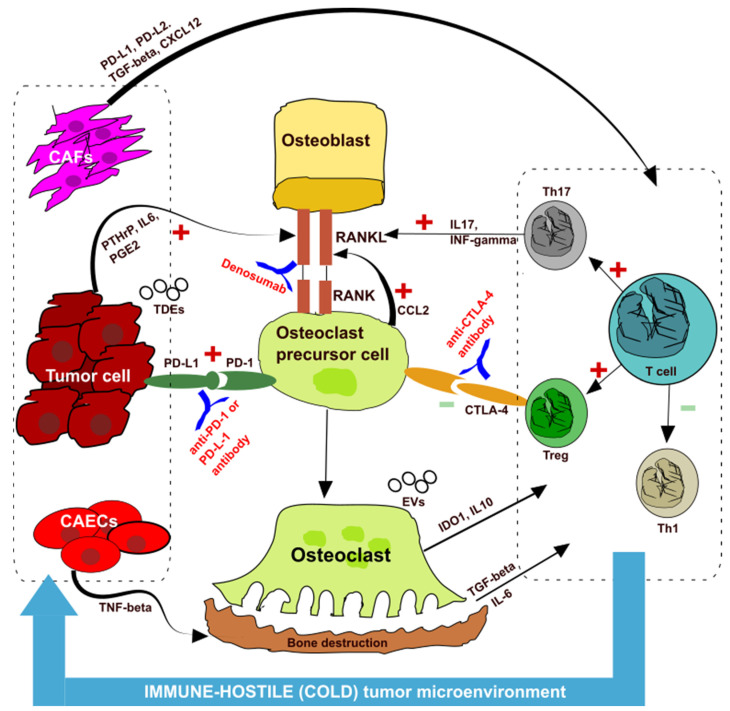
Interaction among the bone, immune system, and cancer cells. CAF: cancer-associated fibroblasts; CAECs: cancer associated endothelial cells; Th: T helper; Treg: regulatory T cells; PD-1: programmed-death 1; PD-L1: PD-ligand (PD-L1); CTLA4: cytotoxic T lymphocyte-associated protein 4; RANK: receptor-activator of nuclear kappaB; RANKL: receptor-activator of nuclear kappaB ligand; PTHrP: parathyroid hormone-related peptide; PGE2: prostaglandin E2; CCL2: chemokine (C-C motif) ligand 2; IDO–1: indoleamine 2,3-dioxygenase-1; IL: interleukin; TGF-beta: transforming growth factor-beta; IFN-gamma: interferon-gamma; TDE: tumor derived exosome; EVs: extracellular vesicles.

Tumor cells themselves release cytokines, which break the balance between osteoblasts and osteoclasts, thereby causing bone resorption and creating an immunosuppressive microenvironment—the so-called “vicious cycle” [19,21] (Figure 1). Cancer cells, in fact, secrete parathyroid hormone-related peptide (PTHrP), prostaglandin E2 (PGE2), and other substances that promote the transformation of osteoblasts into pre-osteoclasts through the receptor-activator of nuclear kappaB ligand (RANKL) pathway, which then promotes differentiation into osteoclasts, causing bone destruction. 

Tumors induce the release of chemokine (C-C motif) ligand 2 (CCI2) from pre-osteoclasts through the PD-1 pathway, which again promotes the formation of osteoclasts by RANKL pathway. So, hypothetically, the action of an anti-RANK antibody such as denosumab could act at this level to break the “vicious cycle”.

Wang et al. [22], in a mouse model of BoM+ lung cancer, had shown that PD-L1 and CCl2 were upregulated and PD-L1 induced osteoclastogenesis. The action of immunotherapy with anti-PD1 or anti-PDL1 drugs could act at this level, blocking the transformation of osteoclast precursor cells in mature osteoclasts. The authors suggest that nivolumab could prevent bone destruction and could alleviate bone cancer pain by suppressing osteoclastogenesis. 

Osteoclasts secrete indoleamine 2,3-dioxygenase-1 (IDO1), interleukin 10 (IL 10), and other substances that induce immunosuppression (Figure 1). The release of transforming growth factor-beta (TGF-beta) due to bone resorption and interleukin 6 (IL6) create an immunosuppressive microenvironment as T cells differentiate into T helper 17 (Th17) and Treg instead of T helper 1 (Th1). This imbalance creates an immune-hostile (cold) tumor microenvironment (TME). Th17 lymphocytes secrete interleukin 17 (IL17) and interferon-gamma (INF-gamma), which again promote osteoclast differentiation.

An important role in this “vicious cycle” is also played by cancer-associated fibroblasts (CAF), which can induce an immunosuppressive microenvironment through PD-1 upregulation and TGF-beta secretion [23] (Figure 1).

Naturally, the RANKL-RANK-OPG (osteoprotegerin) pathway remains the central mechanism of osteolytic metastasis [24,25]. Since RANKL is also expressed in immuno-cells such as NK and T cells, the RANK/OPG balance regulates lymphocyte development in the lymph nodes, maintains dendritic cell activation, and regulates the mediated T response [26]. Recently, the role of soluble RANKL has also been discovered. It can exert a chemotactic activity of the tumor cells in the bone without the involvement of the osteoclasts [24].

In recent years, therefore, the RANKL pathway is considered the link between the bone and the immune system and could be considered a target for improving the efficacy of ICI therapy, as mentioned before [16]. 

The news that has emerged in terms of the interaction between the tumor and the distant site of metastasis relates to the discovery of extracellular vesicles (EVs) [27]. These carry important information that would induce the formation of a pre-metastatic bone niche.

Extracellular vesicles secreted by tumor cells and containing immunosuppressive molecules such as PD-L1 and TGF-beta can be immune escape mediators and may be a possible target for immunotherapy. We know that the PD-L1 secreted by tumor-derived exosomes (TDEs) suppress the activation of T cells in the lymph nodes and promote distant tumor proliferation [28]. While anti-PD-L1 therapy is effective in reducing the immunosuppressive effect on PD-L1 expressing cells, the effect on PD-L1 of TDEs is poor, which may explain why an anti-PD1/PD-L1 therapy is ineffective in some tumors with high PD-L1 expression [29,30] (Figure 1). Nevertheless, to our knowledge, there are no definitive studies on the role of TDEs on bone metastasis; it seems plausible that EVs could impact the efficacy of ICIs in patients with BoM. Other authors hypothesize that exosomes may be involved in the “vicious cycle” by transferring miR-214. This miRNA promotes osteoclast differentiation and mediates intercellular communication between osteoclasts and osteoblasts though an exosomal mechanism [31].

EVs are also involved in the regulation of bone balance. In fact, EVs arising from mature osteoclasts contained RANK. The RANK in EVs may be associated with osteoclast inhibition [32]. Other authors observed that NSCLC-exosomes containing amphiregulin induce EGFR pathway activation in pre-osteoclasts, which in turn causes an increased expression of RANKL [33]. As known, RANKL induces the expression of proteolytic enzymes, triggering a “vicious cycle” in osteolytic bone metastasis.

## 3. Bone Metastasis in NSCLC Treated with Immune Checkpoint Inhibitors

To our knowledge, the impact of all the biological theories listed so far has not been studied in randomized trials, but several retrospective and small prospective data on patients with bone metastasis treated with ICIs are now emerging. We have carefully selected articles, principally regarding clinical observational trials, on the PubMed search engine using the following terms: “bone metastasis in lung cancer”, “osteoimmunoncology”, “immune checkpoint inhibitors and bone metastasis NSCLC”, “pembrolizumab and bone metastasis”, “nivolumab and bone metastasis”, “atezolizumab and bone metastasis”, “nivolumab plus ipilimumab and bone metastasis”, and “bone-targeted therapy and immunotherapy”.

The results of these studies are not always consistent with each other, as is often the case when comparing retrospective or small prospective studies.

Most of the trials would support the hypothesis that NSCLC patients treated with immunotherapy would have a worse prognosis, probably due to the presence of an unfavorable bone microenvironment, as previously mentioned.

One of the first published studies on the concomitant use of immunotherapy and denosumab was the 2018 Liede’s trial [34]. This was an observational study that used Flatiron Health’s HER database from 255 US cancer clinics and included advanced melanoma (n = 66) and NSCLC patients (n = 241) who received denosumab within 30 days of CTLA4 (ipilimumab) or PD1 (pembrolizumab, nivolumab) inhibitors (in NSCLC cohort only 21.6% treated in first-line). The most interesting result of the study is that longer concomitant therapy was associated with increased overall survival, primarily in NSCLC (*p* < 0.0001).

In Zhu’s retrospective Chinese study, a total of 144 NSCLC patients treated with ICI-based strategies from January 2018 to September 2020 were included (59 BoM+ and 78 BoM−) [35]. ICI monotherapy was performed in 27.8% of patients and ICIs combined with chemotherapy/antiangiogenesis was performed in 72.2%. About 40% were treated as first-line. In the multivariate cox regression analysis, the bone metastasis (HR 3.44, 95% CI: 1.97–6.00, *p* < 0.001) as well as ICI monotherapy (HR 1.88, 95% CI: 1.12–3.16, *p* = 0.018) were independently associated with poorer PFS after ICI-based treatment. Similar results were also obtained in the multivariate cox regression for overall survival (HR 3.24, 95% CI: 1.62–6.50 for patients with BoM and HR 2.37, 95% CI: 1.25–4.46 for ICI monotherapy). Another interesting finding was that among the 59 patients with bone metastasis, patients treated with a combination therapy (ICIs + chemotherapy/antiangiogenesis) had a significantly better PFS and OS than those treated with monoimmunotherapy. Only 49% (29 patients) were treated with bisphosphonates. The use of bisphosphonates during ICI treatment significantly prolonged PFS (5.1 vs. 2.1 months, *p* = 0.0039) and OS (17.7 vs. 4.4 months, *p* = 0.02).

Similar results were also seen in other clinical trials. For example, in another Chinese retrospective study, a total of 204 NSCLC patients who received ICI-based treatment from July 2015 to June 2019 were included [36]. Overall, 103 of these patients received ICI monotherapy and 101 ICIs combined with chemotherapy or anti-angiogenesis agents. The study showed a worsening of PFS (4.2 vs. 6.7 months, *p* = 0.0484) and OS (12.5 vs. 23.9 months, *p* = 0.0036) only in the BoM + patients treated with the immunotherapy alone, while there were no significant differences between BoM+ and BoM− patients treated with ICIs associated with chemotherapy. The addition of bone therapies, such as palliative bone radiation (12.5 vs. 16.7 months, *p* = 0.487) and bisphosphonates (12.5 vs. 9.7 months, *p* = 0.568), did not affect OS.

In the Italian study on pretreated NSCLC patients included in the nivolumab expanded access program (EAP), it was confirmed that both PFS and OS in BoM+ patients were reduced regardless of histology [15]. The Italian EAP was a prospective, single-arm, open-label trial conducted in 153 centers from April 2015 to September 2016. In non-squamous patients (1588 total patients), OS was 7.4 months for BoM+ and 15.3 months for BoM− (*p* < 0.0001). In the squamous patient cohort (371 total patients), OS was 5.0 months for BoM+ and 10.9 months for BoM− (*p* < 0.001). In a multivariate analysis, the presence of BoM was independently associated with an increased risk of death (HR 1.5 95% CI: 1.39–1.93 for non-squamous, HR 1.78 95% CI: 1.37–2.31 for squamous). The authors concluded that BoM impairs immunotherapy efficacy, and that accurate bone staging should be included in clinical trials with immunotherapy. Unfortunately, in this large study there was no data on a concomitant use of bone targeted therapy (BTT).

In 2022, a retrospective study exploring the efficacy of pembrolizumab alone or in combination therapy in 110 advanced NSCLC BoM+ patients was published [37]. The trial was conducted from July 2017 to July 2020. Fifty-eight patients (52.7%) received pembrolizumab as a first-line treatment and 52 patients (47.3%) as subsequent lines. The addition of bone therapy, including palliative bone radiotherapy and bone-targeted therapy, increased ORR (34.9% vs. 11.1%, *p* < 0.0001) and prolonged PFS (8.5 vs. 2.0 months, *p* = 0.002). ORR and PFS were significantly improved when bone therapy is combined with pembrolizumab therapy (monotherapy or combination). In particular, BTT alone improved ORR (34.7% vs. 18.4%, *p* = 0.005) and prolonged PFS (8.8 vs. 3.3 months, *p* = 0.003), while palliative bone radiotherapy did not show an increase in either response or survival. The difference in ORR is even greater when bone therapy is added to patients treated with pembrolizumab alone in the first-line (monotherapy 71.4% vs. 25%; combo 43.6% vs. 12.5%).

The retrospective analysis of data on 111 NSCLC patients extrapolated from the prospective Italian register of bone metastasis (BMDB) (from 2014 to 2020) confirmed, despite a limited number of patients treated with ICIs (n = 46), that the use of BTT (30 patients) with ICI monotherapy increases OS from 15.8 months to 21.8 months [38]. Regarding the response on bone evaluated with the MD Anderson criteria, 43.5% of patients obtained a partial response following ICIs plus BTT, while the same response was obtained in only 16.7% of patients treated with ICIs alone. Although not statistically significant, another interesting finding was that patients receiving denosumab had better PFS than patients treated with ICIs alone or with zoledronic acid. Denosumab worked quickly, while the zoledronic acid worked after at least 6 months. 

Another small Japanese retrospective study of 29 patients treated with ICIs from 2016 to 2019 was recently published and confirmed that all patients who were in complete or partial response were treated with the combination of pembrolizumab and denosumab [39].

We have summarized the results of the previous studies cited in Table 1. 

These data suggest that targeting the microenvironment to improve immunotherapy efficacy is a strategy that could be successful.

Several reported data, therefore, support the hypothesis that BTTs increase the activity of ICIs and reverse the negative impact of BoM. Of course, these data would be confirmed in prospective randomized clinical trials.

As pointed out in the previous chapter, the clinical data of these studies could be explained through the “vicious cycle” theory and the presence of an immunosuppressive microenvironment at the bone and primary tumor level (Figure 1). In particular, the release of bone resorption cytokines, such as TGF-beta, increases Th17 suppressor lymphocytes and reduces Th1 effector lymphocytes, creating a cold microenvironment at the level of the primary tumor.

It is not surprising, therefore, that ICIs may be less effective in BoM+ patients.

## 4. Discussion and Future Perspectives

While the majority of breast and prostate cancer patients with bone metastasis are treated with bone targeted therapy, currently only about 50–60% of patients with NSCLC are treated with bone-specific drugs [7,14]. This is due to the fact that, until a few years ago, the prognosis was so severe that it, unfortunately, dissuaded oncologists from prescribing BTT. With the introduction of molecular therapies—but, above all, of immunotherapy—survival has significantly increased. This change of scenario has therefore led to a conceptual shift. First, with the survival gain, the likelihood of developing bone metastasis during the course of the disease also increases. Consequently, more patients may experience SREs. As is known, SREs have a negative impact on the quality of life, so it is necessary to treat most patients with bone resorptive drugs. The second point directly concerns the greater knowledge of the close interconnection between bone marrow and the immune system. In this context, in fact, osteoimmuno-oncology has developed and has hypothesized that adequate treatment of patients with BoM+ and ICI is not only possible but is essential to guarantee them an increase in ORR, PFS, and OS. For these reasons, we believe that there is, or should be, a therapeutic shift in these patients.

The pre-clinical data reported are the result of studies on cellular interactions and pathways involved in the development of osteolytic bone metastasis mainly of breast cancer. Although there are fewer studies in NSCLC, these models are likely to be valid. It is confirmed that the RANK-RANKL pathway is the main pathway responsible for bone resorption. At the same time, more and more evidence demonstrates a close interplay between bone marrow and the immune system (Figure 1). In fact, one system regulates the other.

Therapy with immune checkpoint inhibitors fits into this context. The data available so far demonstrate that bone metastasis is an independent prognostic factor with poor outcomes in patients treated with immunotherapy. In view of the retrospective nature of the clinical data available to us, or the small prospective data, it is clear that there are modest discrepancies in the prognostic impact of ICIs and BTT in these patients. However, most of the trials indicate that the effectiveness of immunotherapy, especially when considered as monotherapy, could be modulated by the tumor and bone microenvironment.

More and more data support the idea that a hostile (cold) tumor microenvironment is created in patients with BoM+, which would reduce the effectiveness of immunotherapy. Some authors believe that the use of bone therapy, in particular bone target therapies, could reverse the resistance to immunotherapy by breaking the “vicious cycle” (Figure 1). Theoretically, an anti-RANK therapy associated with ICIs could be more synergistic. It is clear that larger prospective clinical studies are needed to confirm the hypothesis that emerged from some retrospective studies. 

The clinical studies presented, in fact, have important limits due to the retrospective nature, the sample size of each single study, and the heterogeneity of patients included (first line, subsequent lines, ICI associated or not with other chemotherapeutic/antiangiogenic drugs).

The scope of the association of palliative bone radiation therapy with ICIs in BoM+ patients should also be explored with dedicated prospective studies.

Due to a “cold” TME, patients with BoM may benefit little from the combination of different types of immunotherapies, since ICIs require the presence of effector lymphocytes at the TME level to be effective. While there are drugs that allow the interruption of the “vicious cycle”, transforming the TME from “cold” to “hot” could hypothetically be more promising. For example, bisphosphonates or denosumab that inhibit osteoclast-mediated bone resorption through the inhibition of the pathway of nuclear factor kappa-B ligand receptor (RANKL) could be some of the options. Some chemotherapeutic agents are also capable of reducing immunosuppressive Tregs and inducing apoptosis of myeloid derived suppressor cells (MDSCs). Antiangiogenic drugs, normalizing vascularization, and reducing tumor hypoxia can reduce the production of immunosuppressive cytokines such as IL6, IL10, and IDO.

Thus, the combination of ICIs with chemotherapy, anti-angiogenesis, or bisphosphonates/denosumab could give therapeutic advantages to patients with BoM.

Based on new knowledge, new drug associations could be developed in the future. 

Understanding osteoclast signaling in bone metastasis could help to identify new targets for drug discovery [40], and there are currently drug candidates in different phases of development that are targeting osteoclasts, such as SRC, DKK-1 (dickkopf WNT signaling pathway inhibitor 1), and Sclerostin-targeting compounds [41]. Additionally, interactions with other cell types in the bone metastatic microenvironment could potentially provide new treatment options for bone metastasis [42].

One of the new options for the treatment of BoM is the transfer of gamma-delta T cells. In some studies, mice with breast cancer and BoM+, gamma-delta T cell transfer reduced tumor growth and osteolysis. Promising findings have also been demonstrated in a clinical trial [43]. Another treatment option has been to combine zoledronic acid with IL-2 therapy, which has now been tested in several metastatic tumors [44,45]. 

Other possible positive synergies between anti-CTLA-4 and denosumab and with anti-PD-1 and denosumab have been observed in patients with melanoma BoM+ [46,47].

## 5. Conclusions

Bone is a special immune site with a unique immunosuppressive microenvironment. Bone metastasis impairs immunotherapy efficacy, especially when used alone. Even it is not true in all the trials, bone targeted therapies appear to have a synergistic effect when used in combination with ICIs—maybe due to the interruption of the “vicious cycle”. This action probably restores a less immunosuppressive (“cold” or “hostile”) tumor and bone microenvironment. These promising outcomes have to be confirmed in larger prospective trials—even better if randomized. In view of the new therapeutic scenario of NSCLC, we believe that further studies on the significance of extracellular vesicles and on new therapeutic approaches for bone metastasis are strongly recommended.

## Figures and Tables

**Table 1:** The impact of ICI alone, in combination with chemotherapy (CT-ICI), or bone targeted therapy (BTT), according to different studies. ORR: overall response rate; PFS: progression free survival; OS: overall survival; ↑ increased; ↓ decreased; NV: not evaluable; NR: non reported; * only in patients treated with denosumab.

	Bone Metastasis (BoM)+								
Studies	ICI Alone			CT-ICI			ICI (Mono or Combo) +BTT		
	ORR	PFS	OS	ORR	PFS	OS	ORR	PFS	OS
Liede A [35]	NV	NV	NV	NV	NV	NV	↑	NR	↑
Zhu Y [36]	NR	↓	↓	NR	↑	↑	NR	↑	↑
Li X [37]	=	↓	↓	=	=	=	NR	=	=
Landi L [15]	↓	↓	↓	NV	NV	NV	NV	NV	NV
Qiang H [38]	NV	↓	↓	NV	↑	↑	↑	↑	=
Bongiovanni A [39]	↓	=	↓	NV	NV	NV	↑	↑ *	↑
Asano Y [40]	↓	↓	↓	NV	NV	NV	↑	NR	↑

## Data Availability

Not applicable.
